# The 10/66 Dementia Research Group's fully operationalised DSM-IV dementia computerized diagnostic algorithm, compared with the 10/66 dementia algorithm and a clinician diagnosis: a population validation study

**DOI:** 10.1186/1471-2458-8-219

**Published:** 2008-06-24

**Authors:** Martin J Prince, Juan Llibre de Rodriguez, L Noriega, A Lopez, Daisy Acosta, Emiliano Albanese, Raul Arizaga, John RM Copeland, Michael Dewey, Cleusa P Ferri, Mariella Guerra, Yueqin Huang, KS Jacob, ES Krishnamoorthy, Paul McKeigue, Renata Sousa, Robert J Stewart, Aquiles Salas, Ana Luisa Sosa, Richard Uwakwa, the 10/66 Dementia research group

**Affiliations:** 1 Section of Epidemiology, Health Services Research, King's College London, De Crespigny Park, London SE5 8AF, UK; 2 Facultad de Medicina Finley-Albarran, Medical University of Havana, Cuba; 3 University Policlinic "19 de Abril", Havana, Cuba; 4 Community Mental Health Centre, Marianao, Havana, Cuba; 5 Universidad Nacional Pedro Henriquez Ureña (UNPHU), John F Kennedy Avenue, Internal Medicine Department, Geriatric Section, Santo Domingo, Dominican Republic; 6 Behavioral and Cognitive Neurology Unit, Neuraxis Institute – Neurological Foundation, Buenos Aires, Argentina; 7 University of Liverpool, 6 Stanley Road, Hoylake Wirral, CH47 1HW, UK; 8 Psychogeriatric Unit, National Institute of Mental Health "Honorio Delgado Hideyo Noguchi", José Galvez Barrenechea Avenue # 274. Department 401. Corpac -SAN ISIDRO Lima, Perú; 9 Peking University, Institute of Mental Health. # 51 Hua Yuan Bei Road Haidian District Beijing, 100083, PR China; 10 Christian Medical College, Vellore, India; 11 Srinivasan Centre for Clinical Neurosciences. The Institute of Neurological Sciences, Voluntary Health Services, Taramani, Chennai, India; 12 Public Health Sciences/Molecular Medicine Centre, MRC Human Genetics Unit, University of Edinburgh, UK; 13 Medicine Department, Caracas University Hospital, Faculty of Medicine, Universidad Central de Venezuela, Caracas, Venezuela; 14 The Cognition and Behavior Unit, National Institute of Neurology and Neurosurgery of Mexico, Av. Insurgentes # 3877, Col. La Fama, ZIP Code 14269, Delegacion Tlalpan, Mexico City, Mexico; 15 Dept. of Mental Health, Namdi Azikiwe University Teaching Hospital, Nnewi, Anambra State, Nigeria

## Abstract

**Background:**

The criterion for dementia implicit in DSM-IV is widely used in research but not fully operationalised. The 10/66 Dementia Research Group sought to do this using assessments from their one phase dementia diagnostic research interview, and to validate the resulting algorithm in a population-based study in Cuba.

**Methods:**

The criterion was operationalised as a computerised algorithm, applying clinical principles, based upon the 10/66 cognitive tests, clinical interview and informant reports; the Community Screening Instrument for Dementia, the CERAD 10 word list learning and animal naming tests, the Geriatric Mental State, and the History and Aetiology Schedule – Dementia Diagnosis and Subtype. This was validated in Cuba against a local clinician DSM-IV diagnosis and the 10/66 dementia diagnosis (originally calibrated probabilistically against clinician DSM-IV diagnoses in the 10/66 pilot study).

**Results:**

The DSM-IV sub-criteria were plausibly distributed among clinically diagnosed dementia cases and controls. The clinician diagnoses agreed better with 10/66 dementia diagnosis than with the more conservative computerized DSM-IV algorithm. The DSM-IV algorithm was particularly likely to miss less severe dementia cases. Those with a 10/66 dementia diagnosis who did not meet the DSM-IV criterion were less cognitively and functionally impaired compared with the DSMIV confirmed cases, but still grossly impaired compared with those free of dementia.

**Conclusion:**

The DSM-IV criterion, strictly applied, defines a narrow category of unambiguous dementia characterized by marked impairment. It may be specific but incompletely sensitive to clinically relevant cases. The 10/66 dementia diagnosis defines a broader category that may be more sensitive, identifying genuine cases beyond those defined by our DSM-IV algorithm, with relevance to the estimation of the population burden of this disorder.

## Background

Unlike its predecessor DSM III-R [1], DSM-IV [2] does not specify a criterion for the diagnosis of dementia. However, this can be inferred from the common elements of the DSM-IV criteria for each of the dementia sub-type diagnoses. These are as follows:

### The criteria (A-D) must all be satisfied

A. The development of multiple cognitive deficits manifested by both

1. memory impairment (impaired ability to learn new information or to recall previously learned information)

2. one (or more) of the following cognitive disturbances:

a. aphasia (language disturbance)

b. apraxia (impaired ability to carry out motor activities despite intact motor function)

c. agnosia (failure to recognize or identify objects despite intact sensory function)

d. disturbance in executive functioning (i.e., planning, organizing, sequencing, abstracting)

B. The cognitive deficits in Criteria A1 and A2 each

1. cause significant impairment in social or occupational functioning, and

2. represent a significant decline from a previous level of functioning.

C. The deficits do not occur exclusively during the course of a delirium.

D. The disturbance is not better accounted for by another axis I disorder (for example, major depressive disorder, schizophrenia)

This criterion has been widely used in both clinical and epidemiological research. There is strong face validity. It defines a progressive and relatively pervasive disorder. It seeks to distinguish between dementia on the one hand, and potentially remediable cognitive impairment arising from delirium or mental disorder on the other. Its elements are, for the most part, objectively verifiable. The main weakness is the lack of operational definition. What constitutes memory *impairment*, or cognitive *disturbance*? What is a *significant *impairment in functioning? What represents a *significant *decline in functioning? When is the disturbance *better *accounted for by another axis one disorder? The usual practice is for these questions to be settled according to clinical judgment (in research often by a consensus panel of expert diagnosticians). However, even when structured assessments have been used, the lack of clarity in these areas introduces much scope for unreliability. For the older DSMIII-R criteria, reliability was poorer between as opposed to within international research teams [3]. A WHO assessment of cross-national reliability of application of the DSM-IIR criteria identified worryingly poor reliability for certain elements [4].

The 10/66 Dementia Research Group has as one of its main objectives to compare the distribution and determinants of dementia in different world regions. We have already described an approach for identifying persons with probable dementia in a one phase study design, using a probabilistic predictive algorithm for '10/66 dementia' derived and tested against the criterion of DSM-IV dementia [5]. The algorithm seemed relatively 'education-fair', that is the false positive rate among those with low levels of education was low, and validity was established for a variety of countries and cultures. 10/66 DRG population-based studies are now underway in Cuba, Brazil, Dominican Republic, Venezuela, Mexico, Argentina, Peru, India, China and Nigeria. We also wished to apply DSM-IV criterion directly, and had extended the scope of our one phase assessment to ensure that the necessary data were recorded. The purpose of this paper is to describe our full computerized operationalisation of the DSM-IV criterion, and then to test its validity against local clinician dementia diagnosis and the 10/66 dementia diagnosis, using data from the Cuban population-based study.

## Methods

### Design

We have previously published a comprehensive description of the full protocol for the 10/66 population-based surveys [6]. In the Cuban 10/66 population-based study participants were recruited from eight catchment areas defined by proximity to polyclinics in Havana city and Matanzas. All those aged 65 and over living within the catchment area boundaries were included. Eligible participants were identified from polyclinic registers backed up by systematic door knocking of all households within the catchment area. The DSM-IV dementia and 10/66 dementia survey diagnoses were derived from the structured assessments described below using computerized algorithms, which were run when the full survey was completed. In six of the eight Cuban catchment areas, all participants were interviewed by polyclinic psychiatrists and physicians (one for each catchment area) who were experienced dementia diagnosticians. They were asked to make clinical dementia diagnoses according to DSM-IV criteria, at the end of each individual assessment. At this point, the clinician could not have been aware of the output of the computerized 10/66 and DSM-IV algorithms, and, given their complexity, would have found it difficult to guess the result. This clinical diagnosis was then used as the criterion validation for the computerized DSM-IV algorithm, described below. We also examined the correspondence between the 10/66 Dementia Diagnostic algorithm and clinical diagnosis, and, in the full sample, between the DSM-IV computerized algorithm and the 10/66 Dementia Diagnostic algorithm.

### Measures

The individual cognitive tests used in the 10/66 population-based studies were all validated against a dementia criterion in the earlier pilot phase of the 10/66 programme [5].

1) The Community Screening Interview for Dementia (CSI 'D') [7] consists of a 32 item cognitive test administered to the participant (20 minutes) and a 26 item informant interview, enquiring after the participant's daily functioning and general health (15 minutes). Three summary scores can be generated from the CSI 'D'; a) The cognitive score (COGSCORE), a summary score from the participant cognitive test, with different weightings applied to some items b) The informant score (RELSCORE) an unweighted total score from the informant interview and c) The discriminant function score (DFSCORE), combining COGSCORE and RELSCORE into a single score, using the formula DFSCORE = 0.452322 – (0.01669918*COGSCORE) + (0.03033851*RELSCORE). For the COGSCORE and DFSCORE there are validated cutpoints suggestive of probable and possible cases of dementia.

2) The animal naming verbal fluency task [8] from the Consortium to Establish a Registry for Alzheimer's Disease (CERAD) test battery. Participants are encouraged to name as many different animals as they can in the space of one minute.

3) The adapted CERAD ten word list learning task improved the discrimination of the Hindi Mental State Examination in the Indo-US Ballabgarh dementia study [9]. Six words were taken from the original CERAD battery English language list; butter, arm, letter, queen, ticket, and grass. Pole, shore, cabin, and engine were replaced with corner, stone, book and stick, which were deemed more culturally appropriate [9]. In the learning phase, the list is read out to the participant, who is then asked to recall straight away the words that they remember. This process is repeated three times, giving a total learning score out of 30. Five minutes later the participant is again asked to recall the 10 words, giving a delayed recall score out of 10.

4) The Geriatric Mental State (GMS/AGECAT) [10,11]. This clinical interview generates, from a computerised algorithm (AGECAT) levels of psychopathology (0 = non-case, 1 and 2 = subcase, 3, 4 and 5 = mild moderate and severe case) within nine diagnostic clusters; organic brain syndrome (dementia), schizophrenia, mania, neurotic and psychotic depression, obsessional, hypochondriacal, phobic and anxiety neuroses.

These assessments were supplemented by a structured physical and neurological assessment and an extended informant interview, the History and Aetiology Schedule – Dementia Diagnosis and Subtype (HAS-DDS), a modification of the earlier HAS [12] to focus specifically on information relevant to DSM-IV dementia diagnostic criteria [2], and for dementia subtype diagnosis. With reference to the DSM-IV dementia criteria the HAS-DDS covered the onset and course of the reported cognitive/functional impairment, evidence of impairment in gnosis, praxis and executive function, and the presence or absence of delirium.

Three further assessments not included in either the DSM-IV or 10/66 algorithms were used to test for concurrent validity. These were:

1) activity limitation and participation restriction measured by the WHO-DAS II [13], developed by the WHO as a culture-fair assessment tool for use in cross-cultural comparative epidemiological and health services research;

2) behavioural and psychological symptoms of dementia assessed by the Neuropsychiatric Inventory (NPI-Q)[14];

3) dependency (whether the participant needed no care, some care or much care) ascertained by a series of open-ended questions administered to the informant.

We assessed the severity of dementia in all participants using a computerised operationalisation of the Clinical Dementia Rating (CDR) [15].

### 10/66 dementia diagnosis

10/66 dementia diagnosis is defined as those scoring above a cutpoint of predicted probability of DSM-IV Dementia syndrome [2] from the logistic regression equation developed in the 10/66 international pilot study, using coefficients from the GMS, CSI-D informant and cognitive test interviews and the modified CERAD 10 word list learning tasks [5].

### Operationalisation of the DSM-IV criteria for the computerised DSM-IV algorithm

We settled on a 'top down' approach in which we reviewed the data available in the 10/66 population-based study interview and mapped the information collected onto the DSM-IV criterion applying clinical principles. We sought to operationalise the decision-making process of a competent clinician.

#### Criterion A1 (memory impairment)

Memory was assessed objectively by several items in the CSI'D', which were summed to form a memory subscale, and by the CERAD 10 word list with immediate learning and delayed recall. Three memory scores were thus derived. Additionally, at the end of the GMS interview the interviewer makes an objective rating of memory function. Finally, in the informant section of the CSI'D', the informant is asked about declining memory in general, and the frequency of six specific and characteristic memory lapses; forgetting where s/he has put things, where things are kept, names of friends, names of family, when s/he last saw informant, and what happened the day before.

To define impairment on memory tests, we used the threshold applied in the criterion for mild cognitive impairment; 1.5 standard deviations below the mean for non-demented persons. We differ from the MCI criteria in using education as well as age specific norms. Memory function in older people free of dementia is strongly influenced by level of education, and it seemed important to identify memory impairment only among those performing worst than most of their peers. 1.5 standard deviations below the mean corresponds approximately to the 7th centile of a normal distribution. While it was judged important to set the threshold at the level of mild impairment to ensure sensitivity for detection of early dementia, impaired performance on one test may be explained by extraneous factors; consistent poor performance is much more likely to reflect genuine impairment. In our algorithm, the presence of more unequivocal cognitive impairment on multiple objective tests lowers the degree of corroboration required from the informant report, and vice versa. At the extremes, consistent cognitive impairment on testing means that the criterion is met even in the absence of informant report, and very high levels of informant reported impairment mean that the criterion is met even in the absence of evidence of impairment on formal testing.

#### Criterion A2 (at least one of aphasia, apraxia, agnosia or disturbance in executive functioning)

With the 10/66 assessments, impairment in each of these domains of cognitive functioning can be established through objective cognitive tests, and informant opinion of recent decline.

##### a) Criterion A2a. Aphasia

Both language expression and comprehension are tested in the CSI'D', yielding a six point language subscale. In our pilot study this was somewhat lacking in variance, with a pronounced ceiling effect, and with little effect of age and education. Therefore the same cutpoints were applied to all participants, but as for memory impairment, more convincing evidence of impairment on formal testing lowered the threshold for supporting informant reports, and vice versa.

##### b) Criterion A2b. Apraxia

Praxis was tested by three items in the CSI'D'; taking a sheet of paper, folding it in half and placing it in the lap, and copying interlocking circles and pentagons. These items were used to construct a five point apraxia scale. Two informant report items in the CSI'D' were relevant, those referring to difficulty feeding and difficulty dressing, not explained by physical disability. Here, given the generally poor performance on the praxis test items of those with little education, apparent impairment on formal testing only fulfils the criterion if corroborated by an informant report of at least some degree of impairment

##### c) Criterion A2c. Agnosia

In the CSI'D' the participant is asked to recognize and name four objects (pencil, watch, chair, and shoe) and three parts of the body (knuckle, elbow and shoulder) generating a seven point agnosia scale. In addition, in our extended informant interview, the informant is asked if the participant misidentifies family or friends. The criterion is fulfilled if the informant reports misidentification and/or if two or more errors are made on formal testing.

##### d) Criterion A2d. Disturbance in executive functioning

Executive functioning was tested using the palm-fist-hand test from the Luria battery of frontal lobe tasks, and also by the animal naming test (testing verbal fluency) from the CERAD battery. In the case of the animal naming test, with much of the variance explained by age and education in non-demented persons, we again used 1.5 standard deviations below the age and education norm as the threshold for impairment. In the informant section of the CSI'D' the informant was asked whether the participant experienced difficulty in adjusting to change in routine (frequently or occasionally) and whether there was a change in their ability to think and reason. As part of our extended informant interview we also enquired after difficulty in making decisions, and whether thinking had seemed muddled. Informant reports on these four aspects of executive function were formed into an ad hoc informant scale. Given the lack of domain specificity of the animal naming test (impaired for example by poor concentration) the sub-criterion was not considered to be fulfilled unless impairment in this test was corroborated by some degree of executive impairment reported by the informant. Failure on the palm-fist-hand test was considered sufficient in itself.

#### Criterion B1

This criterion requires that the cognitive deficits in Criteria A1 and A2 each cause significant impairment in social or occupational functioning. Several of the Geriatric Mental State items elicit self-reported memory impairment, including impact on functioning. The participant is asked if memory impairment is a problem for him/her, if they forget names of family or close friends, if they forget where they have placed things, and if they have to make a greater effort to remember things. These items are only rated positive if they occur frequently and cause regular inconvenience. One informant (CSI'D') item is also relevant, where the informant is asked whether memory is a particular problem for the participant. Several CSI'D' items address general impairment in social and occupational functioning likely to have arisen as a consequence of impairment in language, praxis, gnosis or executive function; that is, diminution in range of activities and/or reduced ability to carry out activities, loss of hobby or skills, problems with household chores (not accounted for by physical disability), change in ability to handle money, getting lost inside of the home or in the community. The B1 criterion was considered to be present where the participant or the informant reported memory impairment that was hindering real-life functioning, and the informant reported one or more examples of general social/occupational impairment.

#### Criterion B2

This criterion requires that the cognitive deficits described in A1 and A2 represent a significant decline from a previous level of functioning. In the absence of repeated measures of cognitive function, the only recourse is to the informant history. In the CSI'D' the informant is asked if there has been 'a change in activities', and a 'general decline in mental function'. The HAS-DDS, introduced for the population-based studies to define more precisely course and outcome asks the informant whether overall deterioration, overall improvement or no change has occurred since the onset of the condition, whether there has been gradual decline over a period of two or more years, and whether the intellectual impairment dates from birth or pathology earlier in life. The criterion is established if the informant reports either 'a change in activities', or a 'general decline in mental function' or an overall deterioration or gradual decline over two or more years, provided that the impairment is not considered to date from birth or early life.

#### Criterion C

This criterion requires that the deficits do not occur exclusively during the course of a delirium. Information relevant to this criterion is gathered in the HAD-DDS from an informant; time of onset, type of onset, onset as a result of stroke, presence or absence of clouding of consciousness and confusion worse towards the end of the day. Our algorithm determines that the deficits do occur exclusively in the course of a delirium if the onset was in the last month with sudden onset over 1–3 days, and with either clouding of consciousness (the HAS-DDS item 'changeable over 24 hours, alert at one time, drowsy and confused the next') or confusion worse towards night or evening.

#### Criterion D

This criterion requires that the disturbance is not better accounted for by another axis I disorder (for example, major depressive disorder, schizophrenia). Our algorithm determines that the disturbance is better accounted for by another axis 1 disorder if the stage 2 GMS/AGECAT diagnosis is either schizophrenia or depression, and dementia diagnosis is not confirmed by the 10/66 dementia diagnostic algorithm.

### Analyses

10/66 dementia algorithm cases, and DSM-IV cases are compared with the Cuban clinician interviewers judgment of dementia caseness as an external criterion, with sensitivity, specificity and kappa for agreement.

The prevalence of the DSM-IV sub-criteria and their individual elements is compared between clinically diagnosed dementia cases and high education and low education controls, free of dementia in the 10/66 Cuban population-based study data sets.

10/66 Dementia cases, not confirmed by the DSM-IV computerized algorithm are compared with confirmed DSM-IV cases and controls free of dementia according to the following characteristics; mean CSI'D' COGSCORE and RELSCORE, NPI-Q (behavioural and psychiatric symptoms of dementia) severity and distress scores [14], WHODAS II disability [13], Clinical Dementia Rating (CDR) [15] and the frequency of reports of needing much, some or no care; using independent sample t-tests, and Chi squared tests for trend as appropriate.

## Results

In Cuba, 2909 interviews were completed with 96.4% of all eligible persons responding. All non-response was accounted for by refusals.

In six of the eight Cuban catchment areas, all participants (n = 1887) were interviewed by clinicians who were experienced dementia diagnosticians. 147 (7.8%) were identified by the clinicians as dementia cases, leaving 776 low education controls (completed primary or less) and 958 high education controls (completed secondary or more). After processing our survey data, we identified among these 1887 participants 192 dementia cases according to the 10/66 algorithm (10.2%) and 114 cases according to the computerised DSM-IV dementia algorithm (6.0%). Agreement with the clinician diagnosis was better for 10/66 dementia (Kappa 0.79 [95% CI 0.74–0.83], sensitivity 93.2%, specificity 96.8%) than for the DSM-IV computerised algorithm (Kappa 0.63 [95% CI 0.56–0.69], sensitivity 57.8%, specificity 98.3%). Across the six polyclinic centres, Kappas ranged from 0.63 to 0.86 for 10/66 dementia (median 0.81) and from 0.55 to 0.77 for DSM-IV dementia (median 0.60). According to the CDR severity of the clinically diagnosed cases, the DSM-IV criterion confirmed 86.7% of severe cases, 78.1% of moderate cases, 65.4% of the mild cases and none of the questionable cases. The 10/66 algorithm identified 100.0% of severe cases, 100.0% of moderate cases, 98.1% of mild cases and 77.4% of questionable cases.

Seventy-one percent of the clinically diagnosed cases met the A1 memory impairment criterion, an essential requirement for a DSM-IV diagnosis, 95% had impairments in one or more other domains of cognitive function (A2). All of the sub-criteria for cognitive impairment (A1 and A2) had a very low prevalence in the control groups, suggesting high specificity (Table 1). The only exception was that of the palm-fist-hand test from the Luria battery testing executive function which was frequently 'failed' by those free of dementia. Consequently, more than one third of low education controls and nearly one fifth of high education controls also met the A2 sub-criterion. However, this had little impact on the overall specificity of the A criterion given the high specificity of the memory sub-criterion. 85% met DSM-IV sub-criterion for social and occupational impairment (B1), and 82% met sub-criterion for decline (B2) (Table 2). Combining these two elements, 73% of clinically diagnosed cases met the B criterion compared with around one tenth of high and low education controls. No participants were identified as suffering from delirium (Table 3). Only 1% of the clinically diagnosed cases were classified by the DSM-IV algorithm as 'better accounted for by another Axis 1 disorder' (Table 4). The distribution of DSM-IV sub-criteria by 10/66 dementia caseness was very similar to that previously observed for the clinician diagnoses (Table 5).

**Table 1:** Operationalised criteria for memory impairment (A1), and other domains of cognitive impairment (A2), when applied to the Cuban population based study data set, by clinical dementia status

	Prevalence (%) in those with clinically diagnosed dementia	Prevalence (%) (high education controls)	Prevalence (%) (low education controls)
***A1 Memory impairment (must be present)***			

A1_1 > 1.5 standard deviations below the mean for their age and education group (among those free from dementia) on all three tests of memory and/or	37	2	2
A1_2 > 1.5 standard deviations below the mean for their age and education group on at least two tests of memory and at least two memory lapses reported by informant, of which one should occur 'frequently' and/or	55	3	3
A1_3 > 1.5 standard deviations below the mean for their age and education group on at least one test of memory and at least three memory lapses reported by informant, of which at least two should occur 'frequently' and/or	69	3	4
A1_4 >= four memory lapses are reported by informant, of which at least three occur 'frequently' and/or	78	4	5
A1_5 Severe memory impairment rated by the GMS interviewer	51	1	2
A1 OVERALL	71	3	3

***A2 – language impairment, apraxia, agnosia and executive impairment – at least one must be present***			

*A2a. Language impairment*			

Score of <= 4 on CSI'D' language subscale and/or	32	2	2
Score of <= 5 on CSI'D' language subscale and informant reports that participant 'sometimes' or 'frequently' uses wrong words or has difficulty saying words, and/or	22	1	1
informant reports that participant 'frequently' uses wrong words or has difficulty saying words	23	1	1
A2a OVERALL	52	2	3

*A2b. Apraxia*			

Unable to dress, or wrong sequence/forgets items (DRESS >= 2) and/or	22	0	1
Cannot eat cleanly with proper utensils (FEED >= 1) and/or	20	1	1
Apraxia score <= 3 and some impairment in dressing or feeding (DRESS or FEED >= 1)	29	1	2
A2b OVERALL	31	1	2

*A2c. Agnosia*			

the informant reports that the participant misidentifies family or friends and/or	27	1	1
five or fewer of the seven objects/body parts are named correctly	33	2	4
A2C OVERALL	43	3	5

*A2_d Executive function*			

participant cannot learn palm-fist-hand after five trials and/or	52	7	15
participant cannot sequence palm-fist-hand and/or	69	9	25
informant reports that participant 'frequently' has difficulty in adjusting to routine and/or	25	7	8
informant reports any degree of impairment in three or more of the four examples of executive function	52	2	3
informant reports some degree of impairment in two or more of the four examples of executive function and the participant scored more than 1.5 standard deviations below the mean for their age and education group (among those free from dementia) on the animal naming test	46	1	1
A2_d OVERALL	93	16	31

**Table 2:** Operationalised criteria for impairment in functioning (B1), and decline from a previous level of functioning (B2) when applied to the Cuban population based study data set, by clinical dementia status

	Prevalence (%) in those with clinically diagnosed dementia	Prevalence (%) (high education controls)	Prevalence (%) (low education controls)
***B1 criterion requires that the cognitive deficits in Criteria A1 and A2 each cause significant impairment in social or occupational functioning***			

Memory impairment reported by informant, or	77	7	11
problems in two or more memory areas by informant report at least one of which must be 'frequent', or	90	26	32
any significant self-reported memory impairment (Q341, Q351, Q352 or Q361 >= 1)	69	65	66
Any of the above memory impairments, and	95	67	69
General impairment – one or more informant reported deficits (reduced activities, loss of hobby or skills, problems with household chores, change in ability to handle money, gets lost in the community or at home)	88	11	16

***B2 criterion requires that the cognitive deficits described in A1 and A2 represent a significant decline from a previous level of functioning***			

B2_1 a 'change in activities', and/or	68	7	11
B2_2 a 'general decline in mental function' and/or	80	6	9
B2_3 an overall deterioration and/or	83	5	7
B2_4 gradual decline over two or more years, and	71	4	6
B2_5 the impairment is not considered to date from birth or early life	100	100	100

**Table 3:** Operationalised criteria for delirium (C) ^1^, when applied to the Cuban population based study data set, by clinical dementia status

	Prevalence (%) in those with clinically diagnosed dementia N = 130	Prevalence (%) (high education controls) N = 50	Prevalence (%) (low education controls) N = 95
***C criterion requires that the deficits do not occur exclusively during the course of a delirium***			

C_1 no onset in the last month, and	100	98	100
C_2 no sudden onset in 1–3 days, and	93	94	93
C_3 not changeable over 24 hours, alert at one time, drowsy and confused the next, or	75	85	87
C_4 no confusion worse towards night or evening	52	70	61

1. Only ascertained among those with a current episode characterized by functional and cognitive decline

**Table 4:** Operationalised criteria for alternative cause (D), when applied to the Cuban population based study data set, by clinical dementia status

		Prevalence (%) in those with clinically diagnosed dementia	Prevalence (%) (high education controls)	Prevalence (%) (low education controls)
***D criterion requires that the disturbance is not better accounted for by another axis I disorder (for example, major depressive disorder, schizophrenia)***				

D-1 No AGECAT stage 2 diagnosis of 'schizophrenia' or	Clinical	99	100	100
D_2 No AGECAT stage 2 diagnosis of 'depression' and	Clinical	93	74	67
D_3 Dementia diagnosis confirmed by 10/66 algorithm	Clinical	97	2	4

**Table 5:** Summary of performance of DSM-IV Algorithm in Cuban population based data set, when applied to the Cuban population based study data set, by dementia status according to clinical diagnosis and 10/66 dementia algorithm

DSM-IV criterion	Dementia diagnosis	Prevalence (%) in those with dementia	Prevalence (%) (high education controls)	Prevalence (%) (low education controls)
Criterion A1: Memory impairment	Clinical	71	3	3
	10/66	71	2	2
Criterion A2: Impairment in other cognitive function	Clinical	95	17	33
	10/66	96	17	37
Criterion A (A1+A2)	Clinical	70	2	3
	10/66	70	1	1
Criterion B1: Impaired social/occupational functioning	Clinical	85	9	14
	10/66	90	10	14
Criterion B2: Decline from a previous level of functioning	Clinical	82	10	14
	10/66	85	12	15
Criterion B B1+B2	Clinical	73	7	9
	10/66	79	8	8
Criterion C: Deficits do not occur only in delirium	Clinical	100	100	100
	10/66	100	100	100
Criterion D: Disturbances not better accounted for by another axis 1 disorder	Clinical	99	74	67
	10/66	n/a	71	65
OVERALL (A+B+C+D)	Clinical	58	1	2
	10/66	58	0	0

In the full sample of 2909 participants, 181 of the 315 10/66 dementia cases (58%) were confirmed as cases by the computerised DSM-IV algorithm. Compared with 10/66 dementia cases not meeting DSM-IV criteria, those with DSM-IV dementia were more cognitively impaired, had more cognitive and functional impairment according to the informant, were more disabled, had more Behavioural and Psychological Symptoms of Dementia and more consequent caregiver distress, and greater needs for care (Table 6). The Clinical Dementia Rating severity of the DSM-IV cases was also more severe – 0.5% had questionable dementia, 43.9% mild dementia, 31.2% moderate dementia and 24.3% severe dementia, compared with 50.8% questionable, 34.1% mild, 9.1% moderate and 6.1% severe for the 10/66 cases not confirmed by DSM-IV. However, the 10/66 dementia cases that were not confirmed by DSM-IV were much more similar in all of these respects to the DSM-IV cases than to the non-demented controls. Using Scheffe's test for multiple group comparisons and Kruskal Wallis (non-parametric test based on group ranks), all group differences were statistically significant at p < 0.001 (Table 6).

**Table 6:** Concurrent validation of 10/66 Dementia and DSM-IV dementia diagnoses

		Group 1 (n = 2587) Non-cases	Group 2 (n = 134) 10/66 cases not confirmed by DSM-IV	Group 3 (n = 188) DSM-IV cases	Mean difference (group 2 vs group 1)	Mean difference (group 2 vs group 3)
CSI'D' COGSCORE (cognitive test score)	Mean (95% CI)	30.6 (30.5–30.6)	22.4 (21.3–23.7)	17.1 (15.9–18.3)	-8.1 (-6.9 to -9.3)	5.4 (3.7 to 7.1)
	Median (IQR)	31.0 (29.6–32.1)	23.9 (20.7–27.2)	16.6 (12.3–23.4)		
CSI'D'RELSCORE(informant reports of cognitive and functional decline)	Mean (95% CI)	1.1 (1.0–1.2)	8.3 (7.3–9.3)	15.4 (14.5–16.3)	7.2 (6.2 to 8.2)	-7.1 (-5.8 to -8.5)
	Median (IQR)	0.5 (0.0–1.5)	7.5 (5.5–10.0)	14.3 (10.0–20.0)		
NPI-Q Severity (severity of behavioural and psychological symptoms)	Mean (95% CI)	2.0 (1.9–2.1)	5.0 (4.1–5.9)	7.1 (6.3–7.9)	3.0 (2.2 to 3.9)	-2.1 (-0.9 to -3.2)
	Median (IQR)	1 (0–3)	3 (1–8)	7 (2–11)		
NPI'Q' Distress (distress caused to the caregiver by BPSD)	Mean (95% CI)	1.7 (1.6–1.9)	5.0 (3.9–6.1)	7.3 (6.3–8.4)	3.3 (2.2 to 4.3)	-2.3 (-0.8 to -3.8)
	Median (IQR)	0 (0–2)	3 (0–7)	5 (1–12)		
WHODAS II Disability	Mean (95% CI)	9.6 (9.1–10.2)	36.0 (30.8–41.2)	48.7 (44.0–53.3)	26.4 (21.2 to 31.6)	-12.7 (-5.7 to -19.7)
	Median (IQR)	2.8 (0.0–13.9)	26.4 (8.3–63.2)	50.0 (19.4–77.8)		
Needs for care	No care needed	96.6%	58.7%	29.8%	Chi sq = 1043, 1 df, p < 0.001	
	Needs care some of the time	1.9%	11.6%	15.2%		
	Needs care much of the time	1.4%	29.8%	55.1%		

## Discussion

The sub-criteria for DSM-IV dementia, derived from a computerised algorithm were plausibly distributed in dementia cases (both those identified by clinician diagnosis, and by the 10/66 dementia algorithm) and non-cases. Over 80% of cases met the key A1, A2, B1 and B2 sub-criteria, the sole exception being that of A1, met by 71% of 10/66 algorithm cases. The requirement that each of the A1, A2, B1 and B2 sub-criteria are met accounts for the relatively low proportion, 58%, of clinically diagnosed dementia cases meeting all elements of the computerised DSM-IV criterion. On the one hand the stringency of this approach ensures high specificity; on the other, misclassification error will tend to accumulate reducing the sensitivity of the overall DSM-IV algorithm. We acknowledge a potential weakness of our approach in that the clinical diagnosis, the computerised DSM-IV diagnosis and the 10/66 diagnosis were all derived from elements of the same structured clinician assessment. This may have led to an overestimate of their concordance. Formal validation would have required independent clinical assessments of survey participants by expert clinicians, applying DSM-IV criteria but without relying upon our survey assessments to do so; however, this approach would still not address the problem of unreliability in clinician application of the DSM-IV criteria.

We have intentionally avoided describing any of the diagnostic outcomes in this study as a 'gold standard', preferring instead to explore the underlying dementia construct and its measurement through the relationships observed between the three outcomes; the 10/66 dementia and DSM-IV dementia survey diagnoses and the clinician diagnosis. In reality, each has their strengths and their weaknesses. The DSM-IV criteria are the dominant paradigm in current research and clinical practice, but have been critiqued both for the primacy accorded to memory impairment (a necessary component, which is not, however, a prominent early feature of many dementias, for example vascular dementia and frontotemporal dementia) and for the lack of specificity of the secondary cognitive criteria (aphasia, apraxia, agnosia, disturbance in executive functioning) which are arguably of more direct relevance to stroke than to dementia [16]. We are optimistic that our 10/66 dementia diagnosis provides a robust alternative given its careful cross-cultural development and calibration [5]; however, it has not previously been validated in a community setting. Local clinician diagnoses have strong ecological validity, reflecting as they do local clinical practice. Nevertheless, lack of standardisation may lead to problems with reliability and validity. In the absence of an unimpeachable gold standard, our approach has therefore been closer to that of construct validation as described by Cronbach and Meehl [17] than to criterion validation.

What then have we learnt from this exercise regarding the comparative utility of the 10/66 and DSM-IV diagnoses? Comparison of the characteristics of 10/66 Dementia Algorithm cases confirmed and not confirmed by our computerised DSM-IV algorithm does raise some questions regarding the sensitivity of the DSM-IV criterion, in that many unconfirmed 10/66 cases were still grossly impaired compared with the controls who met neither set of criteria. Furthermore, in Cuba, clinician diagnoses matched more closely to our 10/66 dementia category than to the more restrictive DSM-IV computerised criterion. In principle, the clinician diagnoses were made using the DSM-IV criterion. Clinicians may be less stringent in the thresholds they set for clinical significance, and less rigorous than a computerised system in the application of the algorithm. In either event, our Cuban data tends to suggest that clinically relevant dementia may be prevalent beyond the confines of the narrowly defined DSM-IV criterion when, as in our study, it is strictly applied using a fully-operationalised computerised algorithm. One of the few population-based studies to examine this issue directly, the Canadian Study of Health and Aging [18] reported a prevalence of 20.9% for those aged 65 and over according to clinical consensus compared with 13.7% according to DSM-IV criterion. Mild cases were selectively excluded by the DSM-IV criterion. We found the same. This may have important implications for previous assessments of the global prevalence of dementia [19,20], relying as they do, to a large extent, upon studies that have used the DSM-IV criterion. The more inclusive 10/66 dementia diagnosis may help to establish the true population burden of the dementia syndrome. Conversely, our 10/66 Dementia algorithm may be overdiagnosing dementia. Our earlier pilot study to develop and test the 10/66 algorithm against a DSM-IV criterion, suggests that this is possible; we achieved very high sensitivity (94%), but with a 6% false positive rate in low education controls and 3% FPR in high education controls. We will examine these issues carefully in each of the 10/66 population-based study centres. The relative validity of the two computerised diagnoses used in the 10/66 survey can probably best be settled when their predictive validity is addressed in the forthcoming incidence phase of the 10/66 investigations. Those with dementia would be expected to have declined further, or to have died. Stability or improvement in cognitive function and functional status would argue against the validity of the case definitions.

## Conclusion

We have at least partly established the validity of the computerised DSM-IV algorithm in Cuba. While we cannot assume that this will extend to other 10/66 centres, core face validity is also strong. Clinical principles have been applied by experienced clinicians in the 10/66 group. The operationalisation, and the basis of the decisions that were taken is transparent. The algorithm is described in this paper, and the full computerised algorithm can be obtained on request from the authors.

## Abbreviations

1066 DRG: Dementia Research Group; AD: Alzheimer Disease; ADI: Alzheimer Disease International; AGECAT: Automated Geriatric Examination for Computerised Assisted Taxonomy; APOE: apolipoprotein E;  BPSD: Behavioural and Psychological Symptoms of Dementia; CERAD: Consortium to Establish a Registry for Alzheimer's Disease; CDR: Clinical Dementia Rating; COGSCORE: CSI'D' cognitive function score; CSI-D: Community Screening Interview for Dementia; DFSCORE: CSI'D' discriminant function score; DSM: Diagnostic and Statistical Manual of Mental Disorders; GMS: Geriatric Mental State; HAS-DDS: History and Aetiology Schedule Dementia Diagnosis and Subtype; NPI-Q: Neuropsychiatric inventory; RELSCORE: CSI'D' informant score; WHO-DAS: World Health Organization – Disability assessment schedule.

## Competing interests

The 10/66 Dementia Research Group works closely with Alzheimer's Disease International (ADI), the non-profit federation of 77 Alzheimer associations around the world. ADI is committed to strengthening Alzheimer associations worldwide, raising awareness regarding dementia and Alzheimer's Disease and advocating for more and better services for people with dementia and their caregivers. ADI is supported in part by grants from GlaxoSmithKline, Novartis, Lundbeck, Pfizer and Eisai.

## Authors' contributions

MP prepared the first draft with the assistance of JLdR. Other authors reviewed the manuscript, provided further contributions and suggestions. All of the authors worked collectively to develop the algorithms and methods described in this paper.

## Pre-publication history

The pre-publication history for this paper can be accessed here:


